# Sinus Plain Film Can Predict a Risky Distance from the Lacrimal Sac to the Anterior Skull Base: An Anatomic Study of Dacryocystorhinostomy

**DOI:** 10.3390/diagnostics12040930

**Published:** 2022-04-08

**Authors:** Kuan-Chung Fang, Ren-Wen Ho, Sheng-Dean Luo, Wei-Che Lin, Ching-Nung Wu, Wei-Chih Chen

**Affiliations:** 1 Department of Otolaryngology, Kaohsiung Chang Gung Memorial Hospital and Chang Gung University College of Medicine, Kaohsiung 83301, Taiwan; oscar80640@gmail.com (K.-C.F.); rsd0323@cgmh.org.tw (S.-D.L.); taytay@cgmh.org.tw (C.-N.W.); 2 Department of Ophthalmology, Kaohsiung Chang Gung Memorial Hospital and Chang Gung University College of Medicine, Kaohsiung 83301, Taiwan; wen6530@adm.cgmh.org.tw; 3 Department of Diagnostic Radiology, Kaohsiung Chang Gung Memorial Hospital and Chang Gung University College of Medicine, Kaohsiung 83301, Taiwan; u64lin@yahoo.com.tw

**Keywords:** aplasia, dacryocystorhinostomy, frontal sinus, lacrimal sac, skull base injury

## Abstract

Background: Removal of the surrounding bone during dacryocystorhinostomy may present a higher risk of skull base injury in patients with frontal sinus aplasia. We used sinus plain films to predict cases with a greater risk of a reduced skull base distance in dacryocystorhinostomy. Methods: Sinus plain films and computed tomography data from patients were retrospectively evaluated. The frontal sinus was classified as normal, hypoplastic, or aplastic according to Waters’ view. Correlations of the frontal sinus roof-supraorbital margin (F-O) and the frontal sinus roof-nasion (F-N) distances on plain film with the closest lacrimal sac-anterior skull base (LS-ASB) distance measured on computed tomography images were assessed. Results: We evaluated 110 patients. In total, 16 (11.8%) patients had frontal sinus aplasia, of whom 6 (2.7%) had bilateral and 10 (9.1%) had unilateral aplasia. Sides with frontal sinus aplasia based on Waters’ view had a shorter median LS-ASB distance than normal or hypoplastic sides. The F-O and F-N distances in Waters’ view were significantly positively correlated with the computed tomographic LS-ASB distance. The F-O margin and F-N distance thresholds for predicting an LS-ASB distance < 10 mm, considered a risky distance, were 11.6 and 14.4 mm, respectively, with sensitivities of 100% and 91.7%, and specificities of 76% and 82.7%, respectively. Conclusions: The LS-ASB distance is closer on aplastic frontal sinus sides. Waters’ view on plain sinus films can provide a fast and inexpensive method for evaluating the skull base distance and sinonasal condition during planning for dacryocystorhinostomy.

## 1. Introduction

The lacrimal sac is the beginning of the nasolacrimal duct, primarily located in a fossa formed by the lacrimal bone and the frontal process of the maxilla. Various pathogenesis may obstruct the nasolacrimal system, including orbital trauma, chronic conjunctivitis, and congenital anomalies [1,2,3,4,5,6], for which dacryocystorhinostomy (DCR) is the current mainstay of treatment [7,8,9]. This procedure includes removal of the surrounding bone and widely opening the lacrimal sac with adequate mucosal anastomosis. DCR is usually performed with an external approach, although it has gradually shifted toward an endonasal endoscopic approach in recent decades. During endoscopic DCR, the insertion of the anterior end of the middle turbinate, the so-called axilla, is an important landmark. A major portion of the lacrimal sac is situated above the axilla of the middle turbinate and a significant part of the sac lies above the common canaliculus [10]. During DCR, removal of a sufficient amount of bone from above the axilla of the middle turbinate is necessary to ensure adequate exposure of the lacrimal sac. Inadequate exposure may cause recurrent stenosis; however, extensive removal of bone from above the axilla of the middle turbinate may increase the risk of injuring the skull base.

DCR has a high success rate with a low complication rate, and these complications are usually minor. However, major complications, such as skull base injury, have been reported during DCR [11,12,13,14,15,16,17]. In DCR, accidental skull base injury could occur during removal of the bony structure surrounding the lacrimal sac. Although this complication is rare, the sequelae are serious, and include meningitis, pneumocephalus, and cerebrospinal fluid (CSF) leakage [11,12,13,14,15,16,17]. Previous reports have suggested that a poorly pneumatized frontal sinus and prior trauma history are risk factors of skull base injury during DCR [11,14]. The degree of frontal sinus pneumatization is highly variable between different individuals and even between different sides in the same individual [18]. Siedlecki et al. reported a trend toward less pneumatized frontal sinus in the upper-lateral direction in the right frontal sinus and in women [19]. Theoretically, poor pneumatization of the frontal sinus will cause the anterior skull base to be closer to the frontal process of the maxilla, where the lacrimal sac is located; however, no previous study has investigated this issue.

Computer tomography (CT) is useful for evaluating sinonasal anatomy and pathologies, including that of the nasolacrimal system and nearby bony structures. However, CT is not routinely performed before DCR due to its related high cost and radiation exposure. Rather, sinus plain film is used as a rapid, low cost, and low radiation exposure examination, with fair accuracy, to identify sinonasal anatomy and pathologies [20,21].

In this study, we investigated the distance between the lacrimal sac and the anterior skull base (LS-ASB) according to the extent of frontal sinus pneumatization. The anatomic relationship between the distance of landmarks on sinus plain films and the LS-ASB distance was analyzed, and we evaluated whether these distances could be used to predict the risk of skull base injury during DCR.

## 2. Materials and Methods

### 2.1. Study Design and Patient Population

This cohort study was approved by the institutional review board (IRB) of Kaohsiung Chang Gung Memorial Hospital (reference number 202001424B0). The requirement for informed consent was waived due to the study design and IRB regulations.

Patients who received imaging examinations with both sinus plain film in Waters’ view and CT, from April 2016 to April 2020, at Kaohsiung Chang Gung Memorial Hospital, Kaohsiung, Taiwan, were retrospectively reviewed. The exclusion criteria included sinonasal tumors or lesions with bony destruction noted on the CT scan of the paranasal sinuses or insufficient image quality to identify the landmarks for measurement. Waters’ view, also termed the occipitomental view, is a radiographic view in which the orbitomeatal line forms a 37° angle with the bucky table surface and the mentomeatal line is perpendicular to the plane of the image receptor. According to Waters’ view, we divided the frontal sinus into three types: normal, hypoplastic, and aplastic frontal sinus. Clinical characteristics, including age, sex, body height, and body weight, were obtained from medical records.

**Figure 1:**
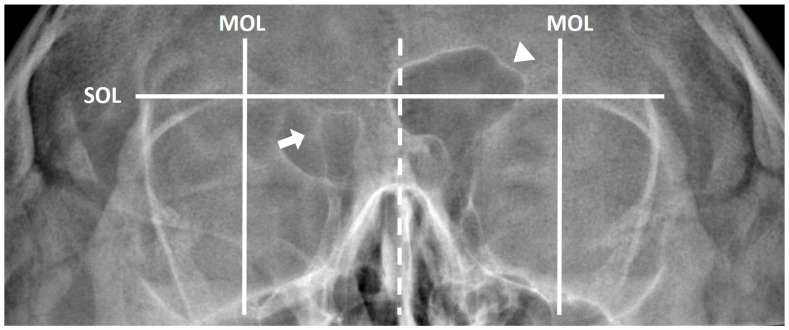
Frontal sinus hypoplasia (arrowhead): a sinus with an oval shape, with the lateral margin lying medial to a vertical line drawn through the middle of the orbit, with a smooth superior margin, and without sinus septa. Frontal sinus aplasia (arrow): absence of frontal bone pneumatization without ethmoid cells extending above the tangential line to the supraorbital margin (orbital roof). SOL, superior orbital line; MOL, middle orbital line.

Frontal sinus hypoplasia was defined as a sinus with an oval shape in which the lateral margin was medial to a vertical line drawn through the middle of the orbit, with a smooth superior margin (Figure 1). Frontal sinus aplasia was defined as a sinus with absent frontal bone pneumatization, without ethmoid cells extending above the tangential line to the supraorbital margin (orbital roof) (Figure 1) [22].

**Figure 2:**
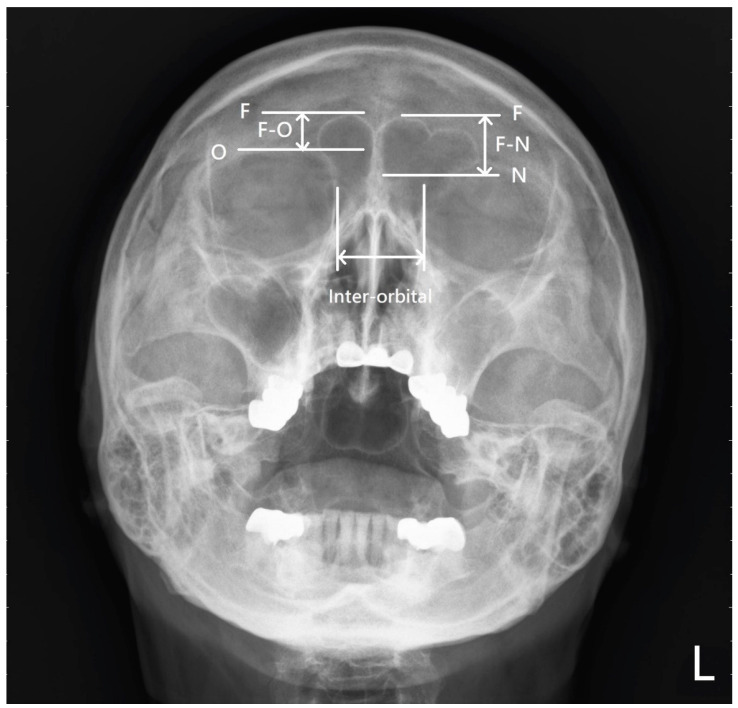
The definition of the frontal sinus roof-supraorbital margin (F-O), frontal sinus roof-nasion (F-N), and inter-orbital distance.

In Waters’ view, the distance between the frontal sinus roof and the supraorbital margin (orbital roof) (F-O) is defined as the distance from the most superior point of the frontal sinus roof to the tangential line of the supraorbital margin. The distance between the frontal sinus roof and nasion (F-N) was defined as the distance from the most superior point of the frontal sinus roof to the tangential line of the most superior point of the nasal bone (frontonasal suture). The inter-orbital distance was defined as the distance between the bilateral medial orbital walls (Figure 2).

**Figure 3:**
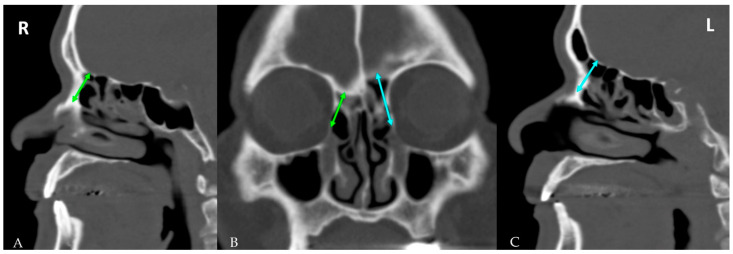
The closest distance between the lacrimal sac and anterior skull base in a parasagittal view (**A**,**C**) and coronal view (**B**) on computed tomography. This distance was shorter in the right aplastic frontal sinus (**A**) than in the left hypoplastic frontal sinus (**C**).

On CT scans, the distance between the lacrimal sac and the anterior skull base (LS-ASB) was measured from the most superior point of the nasolacrimal duct to the nearest anterior skull base (Figure 3).

All the distances in Waters’ view and CT scans were measured using Virtual Place 3D Thin-Client software version 3.4 (AZE Ltd., Canon Medical System, Otawara, Japan). We used the software to find the coordinates of these two points, and then used the formula to calculate the distance between the two points in three dimensions.

### 2.2. Statistical Analysis

We analyzed the correlations among gender, height, weight, inter-orbital distance, F-O, F-N, and LS-ASB. The correlations of LS-ASB in the CT scan with age, height, and weight were analyzed by Spearman’s correlation test. The Mann-Whitney test was used to analyze the distances in Waters’ view and on CT images across the sexes. The F-O, F-N, LS-ASB, and inter-orbital distances between normal and hypoplastic, normal and aplastic, and hypoplastic and aplastic sinuses were statistically compared using the Kruskal-Wallis test.

We used receiver operating characteristic (ROC) curve analysis to determine the F-O and F-N distances in sinus films (Waters’ view) for predicting an LS-ASB distance < 10 mm, which was considered to pose a risk for skull base injury. The area under the ROC curves (AUCs) and 95% confidence intervals (CIs) were used as indexes of accuracy, and the optimal cutoff values were determined from the maximum sum of sensitivity and specificity. *p*-values less than 0.05 were considered to indicate statistical significance. All data were analyzed using SPSS Statistics Version 26 software (IBM Corp., Armonk, NY, USA).

## 3. Results

In total, 110 patients (220 sides) were enrolled in the study. The clinical characteristics of the patients are shown in Table 1. In total, 16 (11.8%) patients had frontal sinus aplasia, of whom 6 (2.7%) had bilateral aplasia and 10 (9.1%) had unilateral aplasia. There were 22 (10%) sides with frontal sinus aplasia, 91 (41.4%) sides with frontal sinus hypoplasia, and 107 (48.6%) normal sinus sides.

The median and interquartile range of the F-O, F-N, inter-orbital, and LS-ASB distances in the normal, hypoplastic, and aplastic groups are shown in Table 2. The sides with frontal sinus aplasia had shorter F-O, F-N, and inter-orbital distances in Waters’ view than those with hypoplastic or normal sinuses (all *p* < 0.001). The sides with frontal sinus aplasia had a closer LS-ASB distance on CT than those with hypoplastic or normal sinuses (all *p* < 0.001). The sides with frontal sinus hypoplasia also had shorter F-O, F-N, inter-orbital, and LS-ASB distances than those with normal sinuses (all *p* < 0.001) (Table 2). The LS-ASB distance on the left side was longer than that on the right side on CT images (left side: 16.3 [14.0–19.2] mm, right side: 14.8 [12.7–17.1], *p* = 0.008). There was no statistically significant difference in F-O (*p* = 0.325) and F-N (*p* = 0.052) between the right and left sides. Males had longer F-O, F-N, inter-orbital, and LS-ASB distances than females (Table 3).

The LS-ASB distance was significantly correlated with F-O (*γ* = 0.604, *p* < 0.001), F-N (*γ* = 0.580, *p* < 0.001), and inter-orbital (*γ* = 0.320, *p* < 0.001) distances. The LS-ASB distance showed no correlation with age but had a weak correlation with height (*γ* = 0.164, *p* = 0.015) and weight (*γ* = 0.269, *p* < 0.001) (Table 4). Multiple linear regression was used to analyze the correlation between the measurements of Waters’ view and CT. The distance of LS-ASB was calculated with the following formula:

*LS-ASB* = −1.893 + 0.285 ∗ *F-O* + 0.516 ∗ *inter-orbital* (*p* < 0.001)

**Figure 4:**
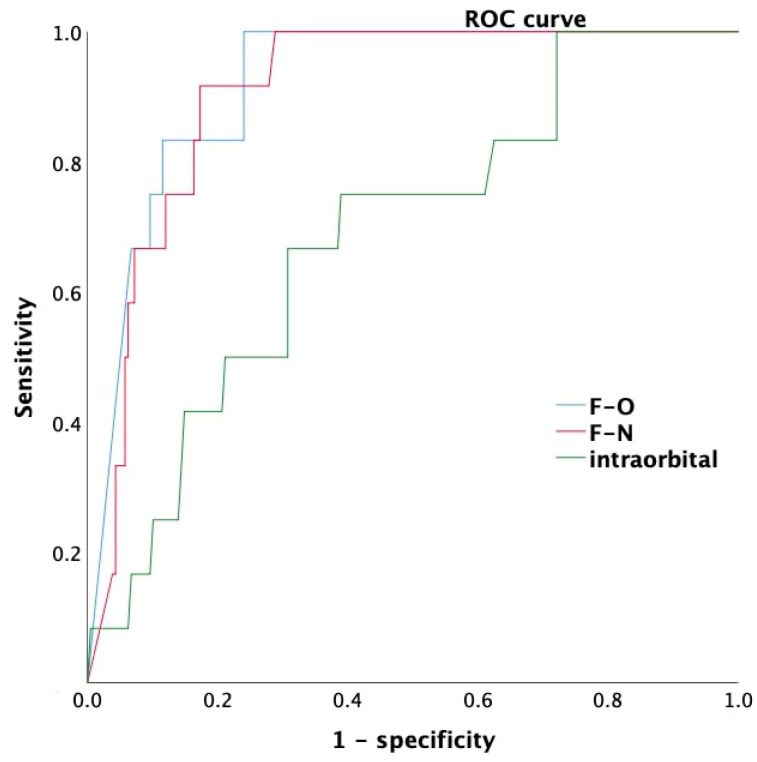
The receiver operating characteristic (ROC) curve showed that frontal sinus roof-supraorbital margin (F-O) and frontal sinus roof-nasion (F-N) distances had greater areas under the receiver operating characteristic curve (AUC).

We used ROC curve analysis to determine the F-N and F-O distances in Waters’ view to predict sides with LB-ASB distances of < 10 mm distance on CT images. The AUCs were 0.92, 0.91, and 0.69 for the F-O, F-N, and intra-orbital distances, respectively (Figure 4). The cutoff values were 11.6 mm for F-O (sensitivity: 100%, specificity: 76%) and 14.4 mm for F-N (sensitivity: 91.7%; specificity: 82.7%) (Table 5).

## 4. Discussion

In this study, we used sinus plain film to predict cases with a greater risk of a reduced skull base distance in DCR. We found that sides with frontal sinus aplasia based on Waters’ view had a shorter median LS-ASB distance than normal or hypoplastic sides. Moreover, the F-O and F-N distances in Water’s view significantly positively correlated with the LS-ASB distance on CT. We also showed that F-O and F-N distances of less than 11.6 and 14.4 mm could sensitively predict a risky distance (<10 mm) to the skull base.

The nasolacrimal duct system is mainly located inside the frontal process of the maxilla and the fornix of the lacrimal sac is situated above the axilla of the middle turbinate [23]. Botek et al. found that the mean distance from the internal common punctum to the anterior aspect of the cribriform plate was 25.1 mm [24]. During external or endoscopic DCR, a Kerrison punch or drill is usually used for osteotomy and to open the lacrimal sac [25,26,27,28]. In some cases, osteotomy is not performed sufficiently due to concerns about injuring the skull base, particularly when inexperienced surgeons perform DCR. On the other hand, excessive osteotomy may increase the risk of skull base injury, particularly in cases at higher risk. Therefore, a thorough understanding of the anatomic relationship between the lacrimal sac and skull base is valuable when planning surgery to prevent skull base injury.

Although DCR, either external or endoscopic, has high success rates and low complication rates, Ali et al. reported a relatively lower success rate and a higher complication rate in less experienced surgeons [27]. Among these complications, CSF leakage was rare but had serious consequences [11,12]. CSF leakage during DCR was explained by either direct or indirect injury to the base of the skull. Indirect damage may be explained by excessive displacement of the middle turbinate or the perpendicular plate of the ethmoid, which are adjacent to the lacrimal fossa, or a spiroid fracture that spreads to the skull base due to the twisting movement of the bone punch [11,24]. Direct damage may occur when the osteotomy is overextended to the anterior part of the skull base, particularly in cases with prior trauma or those with underdeveloped frontal sinuses [11].

Underdevelopment of the frontal sinus, termed frontal sinus aplasia, is present unilaterally in 4.8% and bilaterally in 3.8% of the normal adult population [29]. In theory, a poorly pneumatized frontal sinus will cause the posterior table of the frontal sinus (the anterior skull base) to move anteriorly, closer to the frontal process of the maxilla, where the lacrimal sac is situated. However, no previous study has confirmed this phenomenon. We found that sides with frontal sinus aplasia had a shorter LS-ASB distance than normal or hypoplastic sides. Hypoplastic sides also had a shorter LS-ASB distance than normal sides. The mean LS-ASB distance in our cohort was 11.1 mm (8.4–16.4 mm) in sides with frontal sinus aplasia and 14.6 mm (8.6–28.9 mm) on the hypoplastic sides. Sides with frontal sinus hypoplasia had widely variable distances.

Although CT can offer more detailed information regarding the sinonasal anatomical structure and pathologies, it is expensive, has high radiation exposure, and is not routinely performed before DCR. On the other hand, sinus plain film is rapid, inexpensive, involves less radiation exposure, is more widely available, and is also useful for the evaluation of frontal sinus pneumatization. Among sinus plain films, which include Waters’ view (occipitomental view), Caldwell’s view (occipitofrontal view), and lateral view, Waters’ view is most commonly used for the diagnosis of sinonasal pathologies in clinical practice. It is valuable for diagnosing nasal septal deviation and sinonasal pathologies, particularly in the maxillary and frontal sinuses [22]. The sensitivity of Waters’ view is 70% for the detection of rhinosinusitis and 84.31% for the detection of nasal septal deviation [20]. Therefore, Waters’ view could be used to detect coexisting sinonasal pathologies for comprehensive planning before DCR.

We also found that female patients had shorter F-N, F-O, intra-orbital, and LS-ASB distances than male patients. In the aplasia group, the mean distance was 10.4 mm (8.4–13.6 mm) in female and 12.2 mm (9.4–16.4 mm) in male patients (*p* = 0.043). A previous study reported that the head circumference of males was found to be 1.33 cm larger than that of females [30,31]. We also found a positive correlation between LS-ASB and body height or body weight. This may explain why female patients have shorter distances than male patients. The LS-ASB distance on CT scans was shorter on the right side (left side: 16.3 [14.0–19.2] mm, right side: 14.8 [12.7–17.1], *p* = 0.008). This may be because the right side was predominantly affected with frontal sinus aplasia (63.6%) in our cohort.

In this study, a positive correlation was found between the F-O and F-N distances measured in Waters’ view and the LS-ASB distance measured on CT images (F-O: γ = 0.604, *p* < 0.001; F-N: γ = 0.580, *p* < 0.001). These results indicate that the height of the frontal sinus measured in Waters’ view can be used to predict the LS-ASB distance. Furthermore, we used ROC curve analysis to determine the cutoff values of F-O and F-N for predicting cases with an LS-ASB distance < 10 mm and identified F-O and F-N distances of 11.6 and 14.4 mm, respectively. The AUC and sensitivity values of F-O were superior to those of F-N. Additionally, the F-O distance is easier to measure because the orbital roof usually has a clear outline in Waters’ view. Therefore, we suggest using the F-O distance for predicting the LS-ASB distance. Clinically, surgeons need to be aware that the LS-ASB distance is usually less than 10 mm if the F-O is less than 12 mm; the sensitivity of this prediction was 100% and the specificity was 76% in our cohort, based on ROC curve analysis.

The limitation of this study was that the study was not performed in patients who underwent DCR. Therefore, this was an anatomical study, which cannot truly represent the clinical outcome. However, conducting such an investigation of cases with skull base injuries that occurred during DCR is very difficult due to the rarity of such cases.

In sides with frontal sinus aplasia, the distance from the lacrimal sac to the anterior skull base was significantly shorter than in the normal or hypoplastic frontal sinus sides. There was a significant correlation between the frontal sinus height in Waters’ view and the distance between the lacrimal sac and the anterior skull base in the CT scan. When the F-O distance was less than 12 mm, the distance between the lacrimal sac and the anterior skull base was usually less than 10 mm. Clinically, surgeons should take particular care to prevent skull base injury during DCR in such cases, particularly surgeons with less experience. In conclusion, this anatomical study suggested that Waters’ view can provide a convenient and fast method for predicting the risk of skull base injury during DCR. Thus, Waters’ view on sinus plain films could be included in the preoperative evaluation of patients scheduled for DCR to assess the degree of frontal sinus pneumatization and possible coexisting sinonasal pathologies.

## Figures and Tables

**Table 1:** Demographics of this study cohort.

Characteristic	
Age (year), mean ± SD	48.1 ± 15.2
Gender, *n* (%)	
Male	63 (57%)
Female	47 (43%)
Height (cm), mean ± SD	164.3 ± 9.8
Weight (Kg), mean ± SD	67.4 ± 15.6
Types of frontal sinus	
Right side (*n* = 110), *n* (%)	
Normal	48 (43.6%)
Aplasia	14 (12.7%)
Hypoplasia	48 (43.6%)
Left side (*n* = 110), *n* (%)	
Normal	59 (53.6%)
Aplasia	8 (7.3%)
Hypoplasia	43 (39.1%)

SD: standard deviation.

**Table 2:** F-O, F-N, inter-orbital distance, and LS-ASB in different groups.

Types of Frontal Sinus		F-O (mm)	F-N (mm)	Inter-Orbital (mm)	LS-ASB (mm)
Normal (*n* = 107)	Median (IQR)	18.8 (15.8–21.6)	23.6 (20.3–27.5)	28.7 (26.7–30.1)	17.8 (15.2–20.8)
Hypoplasia (*n* = 91)	Median (IQR)	12.5 (9.5–15.2)	17.4 (13.8–20.6)	26.3 (24.9–28.4)	14.4 (12.7–16.1)
Aplasia (*n* = 22)	Median (IQR)	0.0 (0.0–0.0)	2.4 (0.0–5.8)	24.9 (23.9–27.8)	11.0 (9.4–12.9)
*p* value		<0.001	<0.001	<0.001	<0.001

F-O: distance from the frontal sinus roof to the supraorbital margin (orbital roof); F-N: distance from the frontal sinus roof to the nasion (the most superior point of the nasal bone); LS-ASB, distance between the lacrimal sac and the anterior skull base; IQR: interquartile range.

**Table 3:** The distances in Waters’ view and CT scan in different genders.

	F-O (mm)	F-N	Inter-Orbital	LS-ASB
Male	15.3 (12.5–19.9)	21.2 (17.3–25.9)	28.1 (25.6–29.4)	16.0 (14.0–19.4)
Female	13.4 (8.1–17.8)	17.9 (12.3–23.3)	26.4 (24.3–28.4)	14.8 (12.9–16.9)
*p* value	0.002	0.001	<0.001	0.006

F-O, distance from the frontal sinus roof to the supraorbital margin (orbital roof); F-N, distance from the frontal sinus roof to the nasion (the most superior point of the nasal bone); LS-ASB, distance between the lacrimal sac and the anterior skull base.

**Table 4:** Correlation coefficient between the CT scan, Waters’ view, age, height, and weight.

	F-O	F-N	Inter-Orbital	LS-ASB	Age	Height	Weight
F-O		0.922 *	0.388 *	0.604 *	−0.118	0.236 *	0.214 *
F-N	0.922 *		0.348 *	0.580 *	−0.163 *	0.269 *	0.216 *
Inter-orbital	0.388 *	0.348 *		0.320 *	0.114	0.356 *	0.327 *
LS-ASB	0.604 *	0.580 *	0.320 *		0.117	0.164 *	0.269 *
Age	−0.118	−0.163 *	0.114	0.117		−0.331 *	−0.109
Height	0.236 *	0.269 *	0.356 *	0.164 *	−0.331 *		0.671 *
Weight	0.214 *	0.216 *	0.327 *	0.269 *	−0.109	0.671 *	

* *p* < 0.05; F-O, distance from the frontal sinus roof to the supraorbital margin (orbital roof); F-N, distance from the frontal sinus roof to the nasion (the most superior point of the nasal bone); LS-ASB, distance between the lacrimal sac and the anterior skull base.

**Table 5:** The sensitivity, specificity, and Youden index in F-O and F-N.

	Distance (mm)	Sensitivity	Specificity	Youden Index
F-O	11.2	0.833	0.774	0.607
	11.3	0.833	0.769	0.602
	11.4	0.833	0.764	0.597
	11.5	0.833	0.76	0.593
	11.5	0.917	0.76	0.677
	11.6	1	0.76	0.760 *
	11.6	1	0.755	0.755
	11.7	1	0.75	0.750
	11.8	1	0.745	0.745
	11.9	1	0.74	0.740
	12.0	1	0.736	0.736
F-N	13.4	0.75	0.841	0.591
	13.5	0.75	0.837	0.587
	13.6	0.833	0.837	0.670
	13.8	0.833	0.832	0.665
	14.0	0.833	0.827	0.660
	14.4	0.917	0.827	0.744 *
	14.7	0.917	0.822	0.739
	14.7	0.917	0.817	0.734
	14.8	0.917	0.812	0.729
	14.8	0.917	0.808	0.725
	14.8	0.917	0.803	0.720

* Maximum sum of sensitivity and specificity. F-O, distance from the frontal sinus roof to the supraorbital margin (orbital roof); F-N, distance from the frontal sinus roof to the nasion (the most superior point of the nasal bone).

## Data Availability

Data are available upon reasonable request.
